# Correlation between organizational support, self-efficacy, and core competencies among long-term care assistants: a structural equation model

**DOI:** 10.3389/fpsyg.2024.1411679

**Published:** 2024-09-17

**Authors:** Ankang Liu, Dong Wang, Shanshan Xu, Yixia Zhou, Yao Zheng, Juan Chen, Biyuan Han

**Affiliations:** ^1^Dapeng New District Nan’ao People’s Hospital, Shenzhen, China; ^2^Shenzhen Baoxing Hospital, Shenzhen, Guangdong, China

**Keywords:** long-term care assistants, core competencies, perceived organizational support, self-efficacy, structural equation model

## Abstract

**Introduction:**

Long-term care assistants are taking on more important roles in the healthcare system. The purpose of this study was to investigate what demographic factors influence the core competencies of nursing assistants, as well as to investigate the levels of organizational support, self-efficacy, and core competencies among nursing assistants in China, to explore the relationship between them.

**Methods:**

This is a cross-sectional study with prospective data collection based on a self-report questionnaire. A total of 320 long-term care assistants from two healthcare institutions. We collected socio-demographic characteristics and measured their perceived organizational support, self-efficacy, and core competency levels of the participants. Pearson correlation tests were conducted to examine the relationships among three variables, and a structural equation model was developed to test the interrelationships among these variables.

**Results:**

The results indicated that age, employment type, licensing status, monthly income, pre-job training, and training methods were associated with core competency, with nursing knowledge identified as a weak area in core competencies. There were significant associations among each dimension of perceived organizational support, self-efficacy, and core competencies (*p* < 0.01). The structural equation model demonstrated good fit: *X*^2^/df = 2.486, GFI = 0.974, CFI = 0.988, IFI = 0.988, TLI = 0.977, RMSEA = 0.068, SRMR = 0.013. The direct effect of organizational support on core competencies was 0.37, with self-efficacy mediating the relationship between organizational support and core competencies, yielding an indirect effect of 0.122 and a total effect coefficient of 0.492 (all *p* < 0.001).

**Conclusion:**

Training in core competencies should prioritize nursing knowledge. Enhanced perceived organizational support and self-efficacy among nursing assistants were associated with higher core competencies.

## Introduction

The rapid global aging has significantly increased the need for long-term care services. Forecasts predict that by 2050, the population of individuals aged 60 and over will reach 200 million, or 22% of the global population (WHO, 2022; Newgard and Sharpless, 2013). This demographic shift is expected to drive up the demand for long-term care facilities, as aging often comes with chronic conditions and disabilities. In China, 50% of the seniors have two or more chronic ailments, responsible for 91.2% of deaths in this age group. The WHO (Wang et al., 2022) predicts that by 2050, about 110.5 million people, or 6% of the global population, will need daily care. Of this number, 66 million will be seniors, constituting 59.7% of those requiring daily assistance (WHO, 2015).

Long-term care assistants play a crucial role in addressing hospital staffing shortages, alleviating the caregiving burden on families, and improving the quality of patient care. They provide a range of services, including assistance with feeding, personal hygiene, medical treatments, and rehabilitation activities, monitoring patient health status, and facilitating communication between patients, families, and medical staff (Fryer et al., 2016). The establishment of a specialize nursing assistant group is crucial for improving the quality of life of people with disabilities. However, ensuring the delivery of exceptional care presents significant challenges. Many reports highlight ongoing difficulties in developing this workforce, such as staff shortages, insufficient qualifications, and high turnover rates (Zeng et al., 2019; Li et al., 2022; Kiljunen et al., 2019). China has recently made significant strides in developing its elderly care services industry, including nursing homes and geriatric medical institutions. However, it is important to note that a significant number of nursing assistants do not adhere to uniform certification standards (Song et al., 2014). Challenges regarding the quality and availability of caregivers remain a significant concern (Feng et al., 2012).

Core competency embodies the integration of knowledge, skills, and abilities within an organization that manifest a notable level of proficiency and reliability (Team, 2017). The study by Oeseburg et al. (2015) highlights that care assistants need a wide range of skills to provide professional and high quality care. These include autonomy, management of daily functional health concerns, promotion of healthy aging and well-being, contribution to informal care, collaboration with professional colleagues and active participation in informal care. According to surveys conducted by Bingjie (2021), the core competency of elderly care workers in Xinjiang, China, slightly exceeds the average. However, there is still a need to strengthen training in nursing knowledge and skills. Additionally, the core competencies of nursing assistants are associated with numerous demographic factors. Bingjie (2021) found that in the Xinjiang region in China, the years of employment, professional titles, and whether pre-employment training was attended are closely related to their core competencies. In Taiwan, it has also been reported that age, category of care, and caregiving experience significantly affect core competencies. This is because age can influence the physical and endurance capabilities of nursing assistants (Cheng et al., 2020); those with experience typically possess a higher level of practical knowledge and skills. Beyond these factors, the level of education, employment type, and professional grade of the caregivers are also considered relevant because a higher education level is associated with better reception of nursing education (Li, 2020; Wang et al., 2020); the type of job determines their working environment and income; professional grade tests nursing abilities directly through exams. However, there is still a general lack of related studies, and the demographic factors reviewed are limited. Therefore, one of the objectives of this study is to analyze the demographic factors related to the core competencies of nursing assistants.

Past research has shown perceived organizational support plays a crucial role in the development of employees’ core competencies (Trybou et al., 2014). Perceived organizational support encompasses various aspects, such as providing necessary training resources, fostering a positive work environment, ensuring adequate physical and emotional support, and implementing reward and punishment systems. These supportive measures may enhance caregivers’ professional competencies and increase their job satisfaction and work engagement. Previous studies have reported that nurses’ perceived organizational support is associated with various behaviors, including increased autonomy and commitment (Trinchero et al., 2013), reduced intention to leave (Liu et al., 2018), increased job satisfaction (Ho et al., 2021), and improved nursing practice behaviors (Poghosyan et al., 2013). When employees perceive support from the organization, they are more likely to demonstrate higher levels of core competencies. For instance, regular training and continuing education in the department can help nursing staff acquire the latest medical knowledge and techniques, thereby enhancing their clinical skills (Rochefort and Clarke, 2010; Liu et al., 2016). Additionally, perceived organizational support can provide emotional support. In a supportive work environment, healthcare professionals are better able to cope with work-related stress, reducing the risk of burnout (Spence Laschinger et al., 2009). However, core competency extends beyond nursing practice alone and necessitates specific foundational skills to meet the demands of nursing roles (Oeseburg et al., 2015). To our knowledge, few studies paid attention to nursing assistants’ work environment and perceived organizational support.

Self-efficacy pertains to an individual’s belief in their ability and competence to successfully perform a specific task or behavior (Bandura, 1997). When organizations provide positive support, such as giving constructive feedback and offering opportunities for growth and development, employees’ self-efficacy tends to increase (Garg and Dhar, 2017). The study by Al-Hamdan and Bani Issa (2022) indicated a positive correlation between nurses’ perceived organizational support and self-efficacy, suggesting that higher levels of support in the work environment can lead to increased motivation among nurses. Furthermore, through enhanced organizational support and self-efficacy, individuals may exhibit stronger organizational commitment and better job performance. For instance, a review indicated that organizational support (commitment and emotional commitment) is beneficial for employee outcomes, such as positive emotions and job satisfaction (Rhoades and Eisenberger, 2002). The study by Park and Kim (2023) showed a close correlation between nursing students’ self-efficacy and self-directed learning skills. Social exchange theory explains this process (Cook et al., 2013): the relationship between organizational support and self-efficacy and its impact on job performance can be seen as a reciprocal social exchange process. The theory posits that interpersonal interactions are based on the exchange of mutual benefits, where participants expect their inputs to be reciprocated with equivalent returns. In the workplace, when organizations provide resources, support, and development opportunities, employees typically feel obligated to reciprocate with higher job performance and professional commitment. However, research focusing on nursing personnel has primarily centered on nurses. Due to the many differences between nursing assistants and nurses, their perceived organizational support, self-efficacy, and work competency levels may differ from nurses, but this relationship has yet to be explored.

The objectives of this study are twofold. First, to examine the demographic factors related to the core competencies of nursing assistants. Second, to assess the core competencies, perceived organizational support, and self-efficacy of nursing assistants and to explore the relationships among these factors. The study is based on the social exchange theory, which provides the theoretical foundation for understanding the interactions between employees and organizations. This theory suggests that employees and organizations engage in mutual exchanges, where favorable actions by one party lead to increased dedication from the other (Katou et al., 2020; Jinbao, 2018). In line with this framework, when individuals engage in affirmative actions toward others, such as providing organizational support and ensuring fairness, the recipients of these actions are likely to reciprocate with similar attitudes and behaviors (Cropanzano and Mitchell, 2005; Levinson, 1965). Therefore, the hypothesis for the relationship between perceived organizational support, self-efficacy, and core competencies is:

1. Perceived organizational support relates positively to core competencies.
2. self-efficacy relates positively to perceived organizational support, and positively relates to core competencies.
3. Self-efficacy positively mediates the relationship between perceived organizational support and core competencies.

## Method

### Study design

This is a cross-sectional study.

### Participants and setting

This study used convenience sampling to select participants from two tertiary rehabilitation hospitals in Shenzhen, China. The inclusion criteria for nursing assistants in the study were: (a) being involved in full-time patient care; (b) being 18 years of age or older; (c) having at least 6 months of experience in patient care. The exclusion criteria were: (a) having a family relationship with any of the patients; (b) having a history of mental illness; (c) being unable to understand the content of the questionnaire even after clarification. Following Hoelter’s (1983) guidelines, a sample size of at least 200 is recommended for path analysis to produce statistically reliable results. To meet this criterion, 330 nursing assistants were initially recruited for the study. After removing a number of duplicate responses and quickly completed questionnaires, a total of 320 valid questionnaires remained for research analysis, thus meeting the sample size requirements.

### Instruments

#### Demographic information

The demographic questionnaire was designed independently to collect information on a range of attributes including gender, age, ethnicity, religion, marital status, educational attainment, living area, type of employment, licensed, professional title, years of work experience, monthly earnings, pre-job training, training approach and organizations offering training.

#### Perceived organizational support scale

The questionnaire used in this study was adapted and compiled by Zuo (2009). They found that the homogeneity reliability of the total scale was 0.90 after surveying 480 nurses. This tool has been widely used in China (Huang and Tang, 2024; Ki et al., 2024). It comprises 13 items categorized into emotional support and instrumental support subscale. Each item is rated on a 5-point Likert scale, ranging from 1 (strongly disagree) to 5 (strongly agree), with a total score ranging from 13 to 65. A higher score indicates better organizational support. The Cronbach’s *α* of this scale was 0.964 in this study.

#### General self-efficacy scale

The scale used in this study was developed by Schwarzer et al. (1997) and later translated and revised by Wang et al. (2001). The test results of the scale indicated the internal consistency coefficient was 0.87 and the retest reliability was 0.83, demonstrating good reliability and validity (Wang et al., 2001). The questionnaire consists of 10 items and uses a 4-point Likert scale ranging from 1 (completely disagree) to 4 (completely agree), with a total score of 40 points. Scores below 2, between 2 and 3, and above 3 indicate low, moderate, and high levels of self-efficacy, respectively. A higher score indicates a higher level of self-efficacy. The Cronbach’s *α* of this scale was 0.926 in this study.

#### Core competency

This tool was developed by Jing et al. (2017) specifically for nursing assistants’ core roles and functions. The internal consistency coefficient of the scale was 0.879 and the retest reliability was 0.894. It also showed good reliability and validity in other studies (Cuihong et al., 2024; Renxia et al., 2021). The scale includes 5 dimensions and 27 items: personal qualities (6 items), communication skills (4 items), knowledge of ethics and regulations (6 items), nursing knowledge (7 items), and nursing skills (4 items). Each item is rated on a 5-point Likert scale, with 1 representing “not possessed” and 5 representing “completely possessed.” A higher score indicates stronger core competency for nursing assistants. When the average score of each dimension exceeds 4 (or the total score exceeds 108), it indicates sufficient core competency. The total Cronbach’s *α* of the scale was 0.961 in this study.

### Data collection

This study collected data from nursing assistants working in two hospitals in Shenzhen, China, between November 2023 and January 2024. Data were collected using a mixture of online and traditional methods. The online component used the “Wenjuanxing” platform. To ensure the accuracy and consistency of data collection, three nurses with research experience were selected as data collectors. They were trained on the study objectives, survey content and principles of informed consent before data collection began. The process began after obtaining informed consent from the participants, with the collectors providing guidance to the assistants as needed to complete the questionnaire. Each question was explained in detail and, if necessary, completed by the collectors themselves. Individual support was provided throughout to ensure the quality of responses, and all data collected were subsequently checked by two people for consistency and accuracy.

### Data analysis

After all questionnaires were completed, we integrated the data collected both online and offline into Microsoft Office Excel. SPSS 25.0 (IBM SPSS Statistics for Windows, Version 25.0. Armonk, NY: IBM Corp.) and AMOS Graphics 24.0 (Chicago, ILL: IBM Corp.) were used for data management and analysis. Frequency and percentage were used to describe categorical data, while mean and standard deviation were used to describe continuous data. Independent samples *t*-tests and one-way analysis of variance (ANOVA) were used to compare differences between groups. Pearson correlation analysis was conducted to examine the correlations between variables. The relationships between perceived organizational support, self-efficacy, and core competency were tested using the Structural Equation Modeling (SEM) technique, with the maximum likelihood method. The model is evaluated as good when the path index meets a certain level (Steiger, 2007; Browne and Cudeck, 1993): chi-square (*χ*^2^) *X*^2^/df < 3, goodness-of-fit index (GFI) >0.90, Comparative Fit Index (CFI) >0.90, incremental fit index (IFI) >0.90, Tucker-Lewis Index (TLI) >0.90, Root Mean Square Error of Approximation (RMSEA) <0.08.

## Results

### Basic information

The demographic characteristics of the participants are presented in Table 1. A total of 320 nursing assistants participated in this study. There were 108 males and 212 females. Among them, 163 individuals (50.9%) were aged between 50 and 60 years. The majority, 289 (90.3%), belonged to the Han ethnicity, and 287 (89.7%) reported no religious affiliation. Additionally, 280 (87.5%) were married, and 232 (72.5%) had a high school education. Most participants (62.8%) were contracted employees. Furthermore, 202 (63.1%) held caregiver certificates. Approximately half (48.4%) of the participants did not hold professional titles, and 54.1% had more than 5 years of work experience. Most participants (80.3%) earned a monthly salary ranging from 5,000 to 7,000 yuan, and 80.6% received pre-job training. The predominant training method involved a combination of theory and practice (63.7%), with training primarily conducted by hospitals or departments (56.9%) and companies (31.6%).

**Table 1:** Demographic characteristics of participants (*N* = 320).

Variable	*N* (%)	Total score of core competencies	t/F	P
Gender			0.659	0.51
Male	108 (33.8)	121.19 (13.03)		
Female	212 (66.3)	120.07 (15.16)		
Age; yr., mean (SD)	49.56 ± 8.53		7.031	0.001
≤50	130 (40.6)	121.83 (14.78)		
50 ~ 60	163 (50.9)	120.95 (12.91)		
60 ~ 70	27 (8.4)	110.74 (18.31)		
Ethnicity			10.327	<0.001
Han	289 (90.3)	120.8 (14.32)		
Hui	2 (0.6)	75.5 (4.95)		
Other	29 (9.1)	120.03 (11.26)		
Religion			1.054	0.305
No	287 (89.7)	120.52 (14.17)		
Yes	33 (10.3)	119.73 (17.04)		
Marital status			1.353	0.285
Single	15 (4.7)	109.73 (21.22)		
Married	280 (87.5)	121.07 (13.83)		
Widowed	9 (2.8)	120.11 (21.2)		
Divorced	16 (5)	119.81 (10.43)		
Education level			4.018	0.023
Primary school and below	55 (17.2)	116.44 (17.55)		
Junior high school	232 (72.5)	122.03 (13.02)		
High school/technical secondary school and above	33 (10.3)	116.03 (16.59)		
Living area			2.191	0.115
Urban area	136 (42.5)	121.93 (12.52)		
Suburbs	72 (22.5)	116.99 (17.82)		
Rural	112 (35)	120.87 (14.02)		
Type of employment			4.22	0.018
With staffing	85 (26.6)	121.35 (12.19)		
Independent contractor	201 (62.8)	121.45 (14.37)		
Labor dispatching	34 (10.6)	112.24 (17.75)		
Licensed			3.099	0.002
Yes (certified)	202 (63.1)	122.34 (12.46)		
No (uncertified)	118 (36.9)	117.21 (16.94)		
Professional title				
No	155 (48.4)	119.34 (15.19)	3.43	0.02
Primary	48 (15)	118.46 (13.84)		
Medium level	38 (11.9)	119.53 (17.06)		
Senior	79 (24.7)	124.27 (11.24)		
Working experience			1.373	0.262
1 year and below	13 (4.1)	114.69 (23.43)		
1 ~ 3 years	69 (21.6)	119.61 (14.25)		
3 ~ 5 years	65 (20.3)	118.65 (11.84)		
More than 5 years	173 (54.1)	121.89 (14.53)		
Monthly income (yuan)			3.803	0.023
Less than 5,000	51 (15.9)	116.69 (13.39)		
5,000–7,000	257 (80.3)	121.51 (14.34)		
More than 7,000	12 (3.8)	113.67 (17.96)		
Pre-job training			2.171	0.031
Yes	258 (80.6)	121.3 (13.46)		
No	62 (19.4)	116.89 (17.73)		
Training approach			6.476	0.002
Theory	83 (25.9)	120.45 (13.75)		
Theory and operation	204 (63.7)	121.78 (14.15)		
No training/Self-study	33 (10.3)	112.18 (15.76)		
Organizations offering training (main)			1.033	0.357
Hospital or Department	182 (56.9)	120.68 (14.63)		
Care Company	101 (31.6)	121.19 (14.19)		
Other	37 (11.6)	117.3 (14.35)		

Overall, nursing assistants generally possess enough core competency (total score exceeding 108 points). Univariate analysis results showed that factors such as age, ethnicity, education level, type of employment, licensed, professional title, monthly income, pre-job training, and training methods were associated with the core competency (*P* < 0.05).

### Core competency scores

The scores for different dimensions of core competency are presented in Table 2. Nursing assistants exhibit enough core in five competency dimensions. However, nursing assistants aged 60–70 years fell short of standards in nursing knowledge and nursing skills. Across all dimensions, the average scores were as follows: personal qualities, 4.52 (0.61); communication skills, 4.58 (0.57); knowledge of ethics and regulations, 4.56 (0.57); nursing knowledge, 4.28 (0.69); and nursing skills, 4.42 (0.61) (see Table 3). Notably, nursing assistants scored lower in nursing knowledge compared to other dimensions. This study is not discussing the ethnic differences of the participants in depth due to the small samples size of the Hui participants.

**Table 2:** Scores of dimensions of core competencies and post-hoc test results.

Variable	*N*	VS.	Personal qualities	*P* (2-grp)^a^	Communication skills	*P* (2-grp)^a^	Knowledge of ethics and regulations	*P* (2-grp)^a^	Nursing knowledge	*P* (2-grp)^a^	Nursing skills	*P* (2-grp)^a^
			Mean (SD)		Mean (SD)		Mean (SD)		Mean (SD)		Mean (SD)	
Age; yr., mean (SD)												
≤50	130	50 ~ 60	4.55 (0.63)	0.75	4.61 (0.61)	0.811	4.61 (0.56)	0.607	4.35 (0.7)	0.569	4.49 (0.6)	0.565
50 ~ 60	163	60 ~ 70	4.53 (0.58)	0.121	4.59 (0.51)	0.015	4.58 (0.51)	0.001	4.31 (0.63)	<0.001	4.45 (0.53)	<0.001
60 ~ 70	27	≤50	4.33 (0.63)	0.089	4.31 (0.73)	0.012	4.17 (0.74)	<0.001	3.8 (0.79)	<0.001	3.96 (0.89)	<0.001
Ethnicity												
Han	289	Hui	4.53 (0.59)	<0.001	4.59 (0.56)	<0.001	4.57 (0.55)	<0.001	4.3 (0.69)	0.008	4.43 (0.61)	0.014
Hui	2	Other	2.33 (0.47)	<0.001	2.75 (0.35)	<0.001	2.67 (0.47)	<0.001	3 (0)	0.018	3.38 (0.53)	0.019
Other	29	Han	4.55 (0.51)	0.872	4.56 (0.53)	0.785	4.58 (0.49)	0.899	4.19 (0.66)	0.408	4.41 (0.58)	0.891
Education level												
Primary school and below	55	Junior high school	4.37 (0.71)	0.122	4.4 (0.69)	0.045	4.4 (0.72)	0.104	4.13 (0.83)	0.205	4.33 (0.69)	0.175
Junior high school	232	High school/and above	4.58 (0.56)	0.215	4.65 (0.49)	0.175	4.62 (0.49)	0.133	4.34 (0.64)	0.279	4.46 (0.59)	0.28
High school/and above	33	Primary school and below	4.35 (0.69)	0.994	4.38 (0.77)	0.995	4.37 (0.68)	0.96	4.12 (0.73)	0.54	4.33 (0.58)	0.991
Type of Employment												
With staffing	85	Independent contractor	4.57 (0.57)	0.98	4.57 (0.54)	0.673	4.62 (0.46)	0.965	4.3 (0.58)	0.892	4.45 (0.51)	0.995
Independent contractor	201	Labor Dispatching	4.56 (0.58)	0.026	4.64 (0.52)	0.004	4.59 (0.55)	0.013	4.32 (0.72)	0.035	4.44 (0.64)	0.309
Labor Dispatching	34	With staffing	4.19 (0.75)	0.031	4.18 (0.76)	0.023	4.18 (0.76)	0.009	4.05 (0.75)	0.065	4.25 (0.63)	0.274
Licensed												
Yes (certified)	202	No	4.59 (0.51)	0.006	4.62 (0.53)	0.088	4.65 (0.47)	<0.001	4.34 (0.64)	0.065	4.51 (0.51)	0.001
No (uncertified)	118		4.4 (0.72)		4.5 (0.64)		4.39 (0.66)		4.19 (0.76)		4.28 (0.72)	
Professional title												
No	155	Medium level	4.48 (0.63)	0.996	4.57 (0.59)	0.701	4.48 (0.59)	0.97	4.26 (0.72)	0.865	4.37 (0.67)	0.329
Primary	48	Senior	4.48 (0.54)	0.072	4.46 (0.58)	0.118	4.52 (0.53)	< 0.001	4.15 (0.74)	0.151	4.39 (0.57)	0.059
Medium level	38	Primary	4.42 (0.73)	0.998	4.53 (0.69)	0.584	4.5 (0.72)	0.97	4.29 (0.69)	0.382	4.47 (0.54)	0.53
Senior	79	Primary	4.67 (0.49)	0.248	4.69 (0.46)	0.028	4.76 (0.39)	0.046	4.4 (0.59)	0.051	4.53 (0.53)	0.227
No		Primary		0.98		0.254		0.998		0.335		0.807
Medium level		Senior		0.287		0.148		0.249		0.395		0.667
Monthly income (yuan)												
Less than 5,000	51	5,000 ~ 7,000	4.37 (0.62)	0.027	4.36 (0.65)	0.02	4.45 (0.55)	0.203	4.2 (0.59)	0.342	4.23 (0.6)	0.016
5,000 ~ 7,000	257	More than 7,000	4.57 (0.59)	0.015	4.63 (0.53)	0.267	4.6 (0.54)	0.153	4.31 (0.71)	0.535	4.45 (0.61)	0.625
More than 7,000	12	Less than 5,000	4.14 (0.69)	0.237	4.23 (0.77)	0.93	4.08 (0.82)	0.424	4.18 (0.69)	0.907	4.54 (0.51)	0.11
Pre-job training												
Yes	258	No	4.57 (0.54)	0.003	4.6 (0.55)	0.16	4.59 (0.53)	0.024	4.3 (0.67)	0.457	4.46 (0.57)	0.011
No	62		4.32 (0.79)		4.48 (0.67)		4.41 (0.69)		4.23 (0.78)		4.25 (0.71)	
Training approach												
Theory	83	Theory and operation	4.54 (0.59)	0.693	4.57 (0.55)	0.457	4.52 (0.57)	0.442	4.29 (0.64)	0.701	4.44 (0.61)	0.733
Theory and operation	204	No training/Self-study	4.57 (0.58)	<0.001	4.62 (0.56)	0.005	4.62 (0.53)	0.017	4.32 (0.7)	0.023	4.47 (0.57)	0.001
No training/Self-study	33	Theory	4.15 (0.66)	0.002	4.32 (0.65)	0.035	4.25 (0.7)	0.141	4.03 (0.74)	0.068	4.08 (0.74)	0.003

^aComparison results between the variable column and the VS column.^

**Table 3:** Scores and correlations of perceived organizational support, self-efficacy, and core competencies.

Variables	Range	Mean (SD)	1	2	3	4	5	6	7	8
1. Emotional support	1–5	3.42 (1.00)	1							
2. Instrumental support	1–5	3.74 (1.00)	0.821**	1						
3. Self-efficacy	1–4	3.20 (0.66)	0.383**	0.400**	1					
4. Personal qualities	1–5	4.52 (0.61)	0.359**	0.473**	0.439**	1				
5. Communication skills	1–5	4.58 (0.57)	0.323**	0.390**	0.416**	0.821**	1			
6. Knowledge of ethics and regulations	1–5	4.56 (0.57)	0.336**	0.378**	0.342**	0.761**	0.784**	1		
7. Nursing knowledge	1–5	4.28 (0.69)	0.424**	0.427**	0.384**	0.623**	0.645**	0.731**	1	
8. Nursing skills	1–5	4.42 (0.61)	0.317**	0.309**	0.316**	0.573**	0.582**	0.642**	0.763**	1

**p < 0.01.

In pairwise comparisons, statistical differences were found in communication skills, knowledge of ethics and regulations, nursing knowledge, and nursing skills among different age groups, particularly between the “50–60 years” and “60–70 years” groups, as well as between the “60–70 years” and “≤50 years” groups (“50–60 years” > “60–70 years,” “≤50 years” > “60–70 years”). Disparities in personal qualities, communication skills, and knowledge of ethics and regulations were observed between the “Independent contractor” group and the “Labor Dispatching” group, as well as between the “Labor Dispatching” group and the “With staffing” group (“Independent contractor” > “Labor Dispatching,” “With staffing” > “Labor Dispatching”). Differences in personal qualities, communication skills, and nursing skills between nursing assistants earning RMB 5,000 ~ 7,000 and those earning less than 5,000 were observed (“5,000 ~ 7,000” group > “less than 5,000” group). Nursing assistants with certification and those who received pre-job training showed statistical differences in personal qualities, knowledge of ethics and regulations, and nursing skills compared to those without certification and those who did not receive pre-job training (“certified” > “uncertified,” received training > did not receive pre-job training). Moreover, there were significant differences in all five aspects of core skills between nursing assistants who received “theory and practice” training and those who did not (received “theory + practice” > “self-study,” received “theory + practice” > “self-study”).

### Correlation between perceived organizational support, self-efficacy, and core competency

The scores for perceived organizational support, self-efficacy, and core competency are presented in Table 3. In terms of perceived organizational support, the scores for emotional support and instrumental support were 3.42 (1.00) and 3.74 (1.00) respectively. The score for emotional support was slightly lower than that for instrumental support. Self-efficacy scored 3.20 (0.66). Correlation analyses showed significant associations between each dimension of perceived organizational support, self-efficacy, and core competencies (*p* < 0.01).

### Path analysis

To examine the relationship between the three variables, a structural equation model was constructed, with perceived organizational support as the independent variable, self-efficacy as the mediating variable, and core competency as the dependent variable. The model was optimized using modification indices, demonstrating good fit: *X*^2^/df = 2.486, GFI = 0.974, CFI = 0.988, IFI = 0.988, TLI = 0.977, RMSEA = 0.068, SRMR = 0.013. This study showed a good model fit based on the reference fitting metrics for structural equation modeling presented above.

**Figure 1:**
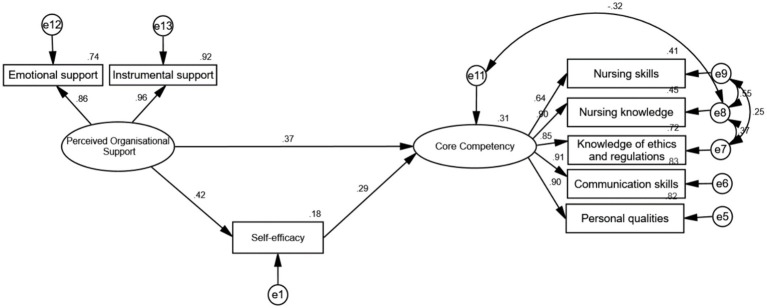
The path among perceived organizational support, self-efficacy, and core competency.

Path analysis indicated that the predictive coefficient of perceived organizational support on self-efficacy was 0.42, and on core competency was 0.37. The predictive effect of self-efficacy on core competency was 0.29. Self-efficacy played a partial mediating role, with an indirect effect coefficient of 0.122 and a total effect coefficient of 0.492 (*p* < 0.001). The path results among the variables are presented in Figure 1 and Table 4.

**Table 4:** The direct, indirect, and total effect results of SEM.

Model pathways	*β* (standardized)	SE	95% CI		P
			LL	UL	
Direct effect					
Perceived Organizational Support → Self-Efficacy	0.423	0.054	0.315	0.522	<0.001
Self-Efficacy → Core Competency	0.289	0.058	0.174	0.402	<0.001
Perceived Organizational Support → Core Competency	0.369	0.045	0.281	0.46	<0.001
Indirect effect					
Perceived Organizational Support → Self-Efficacy → Core Competency	0.122	0.03	0.069	0.187	<0.001
Total effect					
Perceived Organizational Support →Core Competency	0.492	0.041	0.41	0.571	<0.001

## Discussion

The study examined the significant demographic factors influencing core competence and delineated the correlation among perceived organizational support and self-efficacy and core competencies. In particular, self-efficacy emerged as a mediating factor in the relationship between perceived organizational support and core competence.

The average age of participants in this study was 49.56 years, the majority had educational qualifications at or below upper secondary level, and the majority had participated in pre-job training. A survey conducted in Zhejiang Province, China, supports our findings, (Zeng et al., 2019) indicating that education level and pre-job training could predict knowledge of older adults care, while monthly income could predict attitudes toward older adults care. Moreover, participants younger than 50 exhibited advanced competencies in communication, ethics and regulations, and nursing skills, likely a reflection of improved industry standards and the broader training opportunities available to the younger nursing assistants. In addition, nursing assistants in stable employment outperformed those in outsourced positions, possibly because employment instability can hinder talent development and increase job insecurity, leading to less optimal work environments (Burke and Singh, 2016). The findings of these studies provide insights for further practical work. Firstly, optimizing the development of nursing assistants should consider individual differences such as age, educational background, and work experience, and differentiated training strategies can be implemented. Secondly, training involving cutting-edge medical technologies and nursing methods can be designed specifically for younger nursing assistants. Our findings highlight the importance of certification, suggesting that certified care assistants are less likely to neglect older patients. In contrast, those without formal training are more likely to neglect older adults (Wang et al., 2023). This suggests that future efforts should place greater emphasis on the necessity of formal training and certification for nursing staff. Considering the variations in standards across different regions, establishing a unified certification standard remains a challenge (Tyler et al., 2010; Haining, 2023).

Our research showed that, despite their qualifications, nursing assistants have the lower level of nursing knowledge and skills. A major factor contributing to this discrepancy is the lack of systematic training. According to the ‘National Standards for Elderly Care Professionals in China,’ the minimum requirements include a high school education and 180 h of training, which is considered to be quite challenging (Feng et al., 2012). Similarly, a survey in Liaoning Province highlighted gaps in disease care knowledge (Zhou et al., 2022). A study from Taiwan found that nursing assistants excelled in professional, ethical, and legal practices, which correlates with our observations of high scores in ethical regulations and communication skills (Hsieh and Chen, 2017). Unlike our results, health education and literacy were satisfactory in the Taiwanese study, likely due to the participants’ higher education levels. Therefore, continuing education for nursing assistants is essential. In the future, the primary issue targeting the competency enhancement of nursing assistants in China, is still the improvement of nursing knowledge, followed by nursing skills, based on our study’s findings.

In this study, emotional and instrumental organizational support scored at a moderate level. Some research has highlighted the pressures nursing assistants face, including multitasking, clinical emergencies, workplace conflict, resource constraints, and discrimination (Zhang et al., 2023). They also encounter challenges such as heavy lifting, limited autonomy, lack of colleague support, night shifts, and physical assaults (Xiao et al., 2021), impacting their perception of organizational support. Furthermore, the rigid relationship between nurses and nursing assistants may impact the organizational atmosphere and cooperation between them (Potter and Grant, 2004). Conversely, higher perceived organizational support, especially emotional backing (Byrne and Hochwarter, 2008), prompts nursing assistants to contribute more and assume additional responsibilities (Trybou et al., 2014). These results indicated that the emotional and instrumental organizational support for nursing assistants needs to be further strengthened. Managers might consider creating a more inclusive and supportive work environment by improving working conditions, actively addressing workplace conflicts and discrimination, and increasing emotional support, which might allow them to contribute more and assume additional responsibilities.

The self-efficacy scores of nursing assistants in this study were slightly higher than the scores of geriatric care nurses in China (Wang et al., 2023; Zhang and Sun, 2019), which may be related to the workload of caring for patients. Additionally, healthcare professionals may limit contact and care for uncooperative patients (Kritsotakis et al., 2017). Previous research showed that perceived organizational support fosters resources, encouraging employee thriving at work (Abid et al., 2018). This supports the positive association between organizational support and self-efficacy observed in this study, where increased organizational support was associated with better self-efficacy among nursing assistants, which may be conducive for thriving at the workplace (Zhai et al., 2020).

This study revealed that there was a positive correlation between perceived organizational support and core competencies, with self-efficacy mediating their relationship. The study illustrated that enhanced perceived organizational support and self-efficacy were associated with greater potential for nursing assistants to develop core competencies in the workplace. Caesens et al. (2017) posited that employees who perceive their organization as supportive and meeting their social and emotional needs, including a sense of belonging, esteem, appreciation, and recognition, are more likely to be engaged in their work, exhibit greater vigor, and show a greater propensity to acquire new knowledge and skills. In addition, the principle of social exchange suggests that individuals are likely to respond positively to favorable treatment by participating more actively in organizational efforts, thereby improving their job performance (Meira and Hancer, 2021). These insights guide future practical work, emphasizing that organizations should prioritize and boost both the perceived support and self-efficacy of nursing assistants. Management strategies should focus on regular training and development, as well as providing positive feedback and fostering an open, supportive workplace. Such measures not only can enhance employees’ sense of belonging, motivation, and skills but also likely enhance the organization’s overall performance and service quality.

### Limitations

The findings of this study have limitations. Firstly, the sample source is not extensive enough, and future research should incorporate data from multiple centers and larger samples. Secondly, due to the limitations of cross-sectional studies, this research can only reveal correlations between variables and cannot determine causality. Thirdly, the results of this study rely on patient self-reports, which often depend on the recall and reporting of investigators, potentially leading to recall bias.

## Conclusion

This study investigated nursing assistant’ s perceived organizational support, self-efficacy, and core competency levels, which provides evidence for understanding this population and strengthening the weak components. The age, employment type, licensing status, monthly income, pre-job training, and training method are correlated with core competency for long-term care assistants. Nursing knowledge is the weakest aspect of the core competencies and could be strengthened with targeted training. In addition, perceived organizational support and self-efficacy are closely associated with core competencies among nursing assistants. Enhanced perceived organizational support and self-efficacy among nursing assistants are associated with higher core competencies.

## Data Availability

The raw data supporting the conclusions of this article will be made available by the authors, without undue reservation.
